# Identification of a basement membrane-related gene signature for predicting prognosis and estimating the tumor immune microenvironment in breast cancer

**DOI:** 10.3389/fendo.2022.1065530

**Published:** 2022-12-01

**Authors:** Jiehui Cai, Xinkang Zhang, Wanchun Xie, Zhiyang Li, Wei Liu, An Liu

**Affiliations:** ^1^ Department of Chemistry and Chemical Engineering, Hunan Institute of Science and Technology, Yueyang, Hunan, China; ^2^ Department of General Surgery, The Second Affiliated Hospital of Shantou University Medical College, Shantou, Guangdong, China

**Keywords:** breast cancer, basement membrane, prognosis, tumor immune microenvironment, candidate drugs

## Abstract

**Introduction:**

Breast cancer (BC) is the most common malignancy in the world and has a high cancer-related mortality rate. Basement membranes (BMs) guide cell polarity, differentiation, migration and survival, and their functions are closely related to tumor diseases. However, few studies have focused on the association of basement membrane-related genes (BMRGs) with BC. This study aimed to explore the prognostic features of BMRGs in BC and provide new directions for the prevention and treatment of BC.

**Methods:**

We collected transcriptomic and clinical data of BC patients from TCGA and GEO datasets and constructed a predictive signature for BMRGs by using univariate, least absolute shrinkage and selection operator (LASSO) and multivariate Cox regression analysis. The reliability of the model was further evaluated and validated by Kaplan-Meier survival curves and receiver operating characteristic curves (ROC). Column line plots and corresponding calibration curves were constructed. Possible biological pathways were investigated by enrichment analysis. Afterward, we assessed the mutation status by tumor mutational burden (TMB) analysis and compared different subtypes using cluster analysis. Finally, we examined drug treatment sensitivity and immunological correlation to lay the groundwork for more in-depth studies in this area.

**Results:**

The prognostic risk model consisted of 7 genes (FBLN5, ITGB2, LAMC3, MMP1, EVA1B, SDC1, UNC5A). After validation, we found that the model was highly reliable and could accurately predict the prognosis of BC patients. Cluster analysis showed that patients with cluster 1 had more sensitive drugs and had better chances of better clinical outcomes. In addition, TMB, immune checkpoint, immune status, and semi-inhibitory concentrations were significantly different between high and low-risk groups, with lower-risk patients having the better anti-cancer ability.

**Discussion:**

The basement membrane-related gene signature that we established can be applied as an independent prognostic factor for BC and can provide a reference for individualized treatment of BC patients.

## Introduction

Breast cancer (BC), whose number of global new cases is estimated to be 2,261,419 in 2020, is the most common malignant tumor in the world (1). Although BC has made significant progress in risk factors, early diagnosis, and treatment strategy, especially improvements in chemotherapy, targeted therapy, and immunotherapy, its prognosis is still unsatisfactory. Molecular typing, pathological staging, tissue type, treatment, and so on affect the prognosis of BC. In addition, patients with the same molecular type and clinical characteristics have different prognoses, which may be caused by different responses to chemotherapy or immunotherapy (2). It indicates that there may be potential biomarkers to change the tumor microenvironment, thereby affecting the treatment and prognosis of the tumor.

Basement membrane (BM), a sheet-like structure, is a cell-adherent extracellular matrix that is widely distributed in metazoan tissues, lies beneath epithelial cells, and surrounds most tissues. As core structural components of BMs, collagen type IV, laminins, nidogens, and heparan sulfate proteoglycans direct cell polarity, differentiation, migration, and survival (3). For example, it is laminins that are chemotactic for certain tumor cells (4), which contribute to promoting tumor cell growth. The ability of the BMs to control the growth of blood vessels and tumors, maintain the integrity of the skin and neuromuscular structures, and promote adipogenesis and fibrosis has been proven and has gradually become the center of the biological field (3). Based on the diversification and function of BM, variation in its genes is not only considered to underlie human disease (5) but also BM proteins are considered as targets of autoantibodies in immune disorders (6). It is well known to us that all those mutations in laminin cause skin disease due to the disruption of hemidesmosomes. Alport’s syndrome is caused by mutations in type IV collagen. In terms of tumors, dysregulation of BM is the key to tumorigenesis, and the over-proliferation of some tumor cells such as breast cancer, colon cancer, and prostate cancer is closely related to the overexpression of laminin (7). BM is bound up with tumor and non-tumor diseases, as defects in BM protein expression and turnover play key pathogenic roles in cancers, diabetes, and fibrosis (8, 9). Therefore, there is a doubt whether BM is related to the occurrence and development of BC or not. Given the lack of previous studies and reports on BM and BC, to fully understand the impact of BM-related genes on BC, the aspects of gene expression, survival prognosis, gene enrichment analysis, molecular typing, tumor immune microenvironment, and drug sensitivity analysis were explored in the study, which can provide a theoretical basis for further research on the molecular mechanism between BM and BC, and provide a new direction for the prevention and treatment of BC as also.

## Materials and methods

### Data and genes acquisition

We obtained RNA transcriptomic and clinical data required in this study from The Cancer Genome Atlas (TCGA, https://portal.gdc.cancer.gov/) database and Gene Expression Omnibus (GEO, https://www.ncbi.nlm.nih.gov/geo/) database. The TCGA dataset contains 1222 human breast tissue samples, including 1109 BRCA (breast invasive carcinoma) and 113 normal samples. The patients in the TCGA database served as the training set for the prognostic model in this study, from which we extracted basic information, survival data and pathological staging of the patients. The GEO data were selected from the GSE20685 dataset, which contained 327 breast cancer samples with survival information. We obtained a total of 224 basement membrane related genes (BMRGs) for identification through extensive literature reading. The gene transfer format (GTF) was obtained from Ensembl (http://asia.ensembl.org), which was used to distinguish lncRNAs from genes about the samples in the TCGA database.

### Construction of the prognostic signature

We used R software (version: 4.1.2) to analyze the data that had been downloaded and organized. The “Limma” package was used to compare BMRG expression in tumor and normal patients in the TCGA and GEO datasets and to filter for differentially expressed genes (10). The filtering criteria were set to |log2 fold change (FC) | > 0.5 and false discovery rate (FDR)< 0.05. The results were visualized using the “pheatmap” package to create the volcano map and heat map. We then combined selected BMRGs with clinical data and used univariate Cox regression analysis with the “survival” package to screen for genes that were significantly associated with prognosis (*p<* 0.05). The training set (TCGA dataset) and validation set (GEO dataset) together constitute the prognostic cohort, and the prognostic genes co-expressed by TCGA and GEO are screened out. Next, using LASSO regression analysis with the “glmnet” package to further exclude overfitting BMRGs (11), we screened 11 BMRGs strongly associated with BC patients’ prognosis. Lastly, we used multivariate Cox risk regression to analyze the 11 BMRGs and identified 7 BMRGs. Based on it, we constructed the prognostic model. The following equation assessed the risk score of BC patients.


$$\text{Risk score}=\sum_{k=1}^{7}\text{Coef}(k)\times E(k)$$

Coef(k) represents the regression coefficient of BMRGs, and E(k) represents the expression level of BMRGs. We used the median risk score as the cut-off for patients in the high-risk and low-risk groups.

### Internal validation of BMRG prognostic signature

After establishing the prognostic model, it is necessary to validate the resulting model for the purpose of demonstrating its reliability in predicting prognosis. We compared the overall survival (OS) between the two groups based on the Kaplan-Meier log-rank test to see if there was a significant difference. The “pheatmap” package was used to plot risk curves, survival status maps, and risk heat maps for the TCGA and GEO datasets to visualize the effect of BMRGs on the prognosis of BC patients for better observation and comparison. In addition, we performed an independent prognostic analysis using univariate and multivariate COX regression analysis to determine whether the established model was independent of other clinicopathological characteristics as an independent prognosis factor. In this process, we excluded 51 patients with overall survival of fewer than 30 days and 183 patients with missing staging data, including 24 patients without stage, 150 patients without M stage, and 9 patients without N stage. The receiver operating characteristic (ROC) curve was used to reflect the relationship between sensitivity (TPR, true positive rate) and specificity (FPR, false positive rate) at different thresholds, and the area under the curve is called the AUC (Area Under Curve) (12). The larger the area, the higher the accurate value of the prediction. We plotted the ROC curves and calculated the AUC values using the “survminer” and “timeROC” packages.

### Nomograms and calibration curves construction

The Nomogram is based on multivariate COX regression analysis, integrating multiple predictors to express the interrelationship between variables in the prognostic model, and is widely used in oncology (13). We used the “regplot”, “survival”, and “rms” packages to integrate clinicopathological characteristics and risk scores, including age, gender, stage and TMN stage. Nomograms for 1 year, 3 years, and 5 years were created for the TCGA and GEO datasets, respectively. The calibration curves were constructed based on the Hosmer-Lemeshow test, and the accuracy of the nomogram in guiding prognosis was determined by comparing the fitting degree between the actual observations and the predicted results.

### Functional enrichment analysis

We screened differentially expressed genes in different risk groups with |log2 fold change (FC) > 0.5| and with false discovery rate (FDR)< 0.05. The Gene ontology (GO) (14) enrichment of basement membrane-related prognostic genes was analyzed to explore the biological processes (BP), cellular components (CC) and molecular functions (MF). In addition, we used GSEA (Gene Set Enrichment Analysis) (15) to identify the biological pathways of BMRG, using *p*< 0.05 and FDR< 0.05 as the analysis criteria. All biological pathways were obtained from the Kyoto Encyclopedia of Genes and Genomes (KEGG).

### Study of TMB and correlation of prognostic signature

TMB (16) is the total number of mutations per megabase in the tumor tissue. We used a Perl script to obtain each sample’s TMB and mutation frequency in the tumor mutation data coming from TCGA. And all samples in the dataset were divided into high TMB and low TMB groups and combined with the patient’s survival data for analysis. The TMB survival curve was plotted. We plotted waterfall plots for visual analysis of gene mutations in the high and low-risk groups. TMB variance and correlation analyses were further conducted for the high and low-risk groups. Moreover, we performed a combined analysis of high and low TMB and high and low-risk groups to further investigate the effect on patient survival.

### Exploration of tumor immune microenvironment, immune cell infiltration and immune-related functions

It was the Wilcoxon rank sum test that was used for analyzing the differences in StromalScore, ImmuneScore, and ESTIMATEScore between high and low-risk groups. We analyzed the immune microenvironment of the tumor from these three aspects. Simultaneously, we calculated the proportion of immune cell infiltration using various algorithms, including XCELL (17), TIMER (18), QUANTISEQ (19), MCPCOUNTER (20), EPIC (21), CIBERSORT-ABS, and CIBERSORT (22). We visualized it in the form of a bubble plot. Ultimately, we scored the different infiltrating immune cell subpopulations in the samples to assess the changes in immune function in high and low-risk groups and plotted the multi-box plot to visualize the results.

### Cluster analysis of BMRGs

Based on the screened prognostic model of the 7 BCRGs, we performed cluster analysis using BC samples from the TCGA cohort. In the light of “ConensusClusterPlus” R package, we divided tumor samples into different subgroups. The clustering excluded groups with small sample sizes while strengthening intra-group correlations and reducing inter-group correlations. In the meantime, the cumulative distribution function curve was steadily increasing. Next, we plotted survival curves using the “survival” and “survminer” packages to analyze the survival differences of the clustered samples. We also generated Sankey plots using the “dplyr”, “ggplot2”, and “ggalluvial” packages to analyze the relationship between clustering and risk relationship. We used t-distributed Stochastic Neighborhood Embedding (tSNE) to analyze different clusters (23) and high- and low-risk groups to visualize the grouping of clusters. The progression of tumor disease is closely related to the immune microenvironment, so we again analyzed the differences in StromalScore, ImmuneScore and ESTIMATEScore between clusters. Using various algorithms, including TIMER (18), CIBERSORT (22), CIBERSORT-ABS, QUANTISEQ (19), MCPCOUNTER (20), XCELL (17), and EPIC (21), we analyzed the immune infiltration in different clusters and used the “pheatmap” package to generate a heatmap. Eventually, it was the pRRophetic (24) algorithm that was utilized to analyze the maximum inhibitory concentration (IC50) of the drug response data for BC patients under different clustering conditions on Genomics of Drug Sensitivity in Cancer (GDSC) (https://www.cancerrxgene.org/). *p*< 0.001 was considered significant.

### Prediction of potential drug sensitivity and clinical immune efficacy by BMRGs

BC’s research cannot be separated from the development of new drugs. Based on the IC50 of GDSC, we assessed the drug sensitivity of drugs in the dataset using the “pRRophetic” package (24), and the “ggplot2”, “ggpubr”, “limma” and “reshape2” packages were used to analyze statistical differences in the expression levels of common ICI-related immunosuppressive molecules. We assessed differences in drug sensitivity between high and low-risk groups using the Wilcoxon test and plotted box plots, with *p*< 0.001 considered significant.

## Results

### Screening and identification of basement membrane-related genes

**Figure 1:**
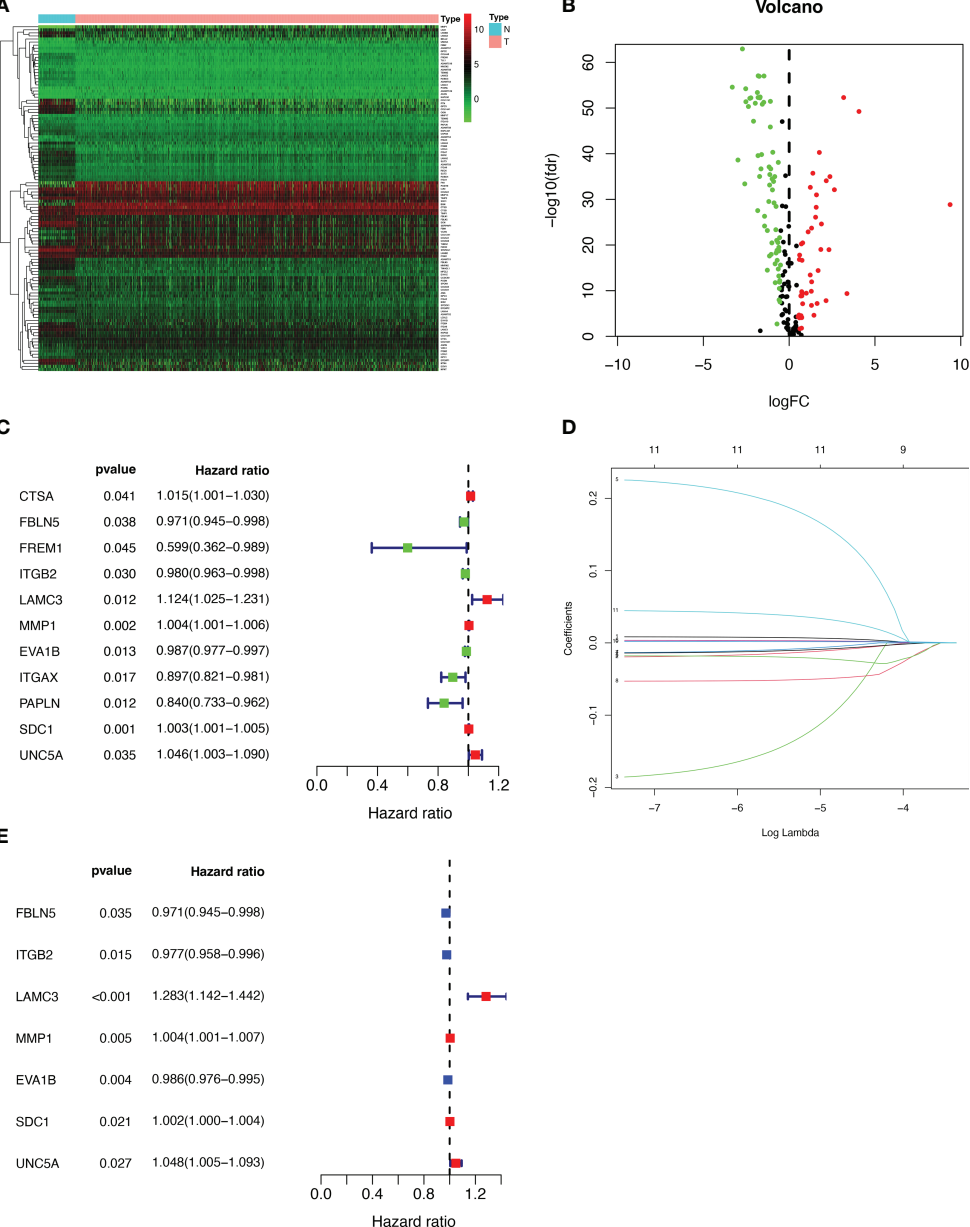
Extraction of differentially expressed BMRGs. **(A)** Heatmap of 113 differentially expressed BMRGs. **(B)** Volcano plot of 113 BMRGs expressed in BC tissues. Red dots represent up-regulated genes in tumor tissue while green dots represent down-regulated genes. **(C)** Forest plot of prognostic BMRGs extracted by univariate Cox regression analysis. **(D)** LASSO regression analysis curve. **(E)** Forest plot of prognostic BMRGs extracted by multivariate Cox regression analysis. BMRGs, basement membrane-related genes.

We extracted 1039 samples based on the TCGA database, after excluding cases with missing or less than 30 days of survival time. By differential analysis of the expression of 224 BMRGs, we identified 113 BMRGs that were differentially expressed in normal and tumor tissues (**Supplementary Table 1**) (|log2 FC|>0.5 and FDR<0.05). The heat map clearly shows the expression of these 113 BMRGs in normal and tumor tissues (**Figure 1A**). According to the volcano plot, 46 of the BMRGs were upregulated and 67 were downregulated in tumor tissues (**Figure 1B**).

### Establishment and validation of the prognostic signature for BMRGs

**Figure 2:**
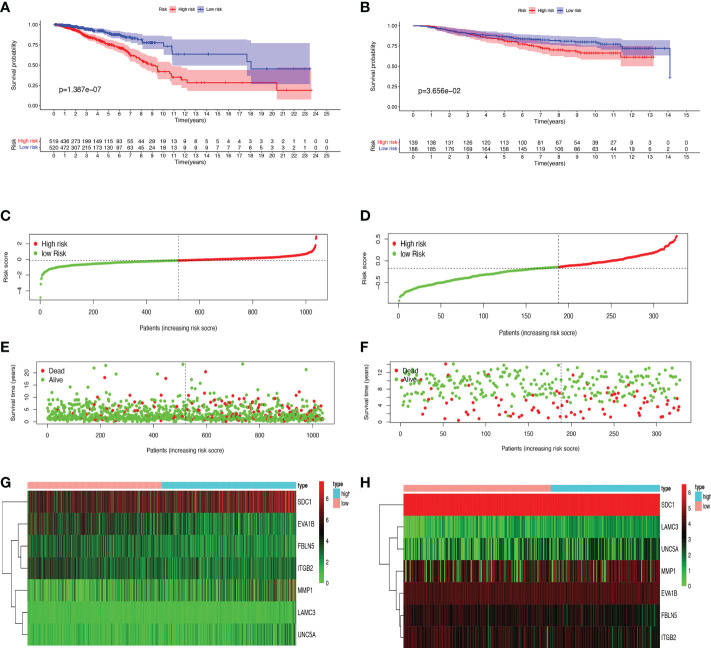
Prognosis and risk score analysis of the 7 BMRGs. **(A, B)** Survival analysis based on the Kaplan-Meier method for OS of BC patients in high- and low-risk groups and plotting survival curves. **(C, D)** Risk score distribution of BC patients. **(E, F)** Scatter plot of BC patients’ survival status. **(G, H)** Risk heat map of 7 BMRGs expressions in the training set **(G)** and validation set **(H)**. **(A, C, E)** Training set (TCGA cohort); **(B, D, F)** Validation set (GEO cohort); BC, breast cancer; OS, overall survival.

**Figure 3:**
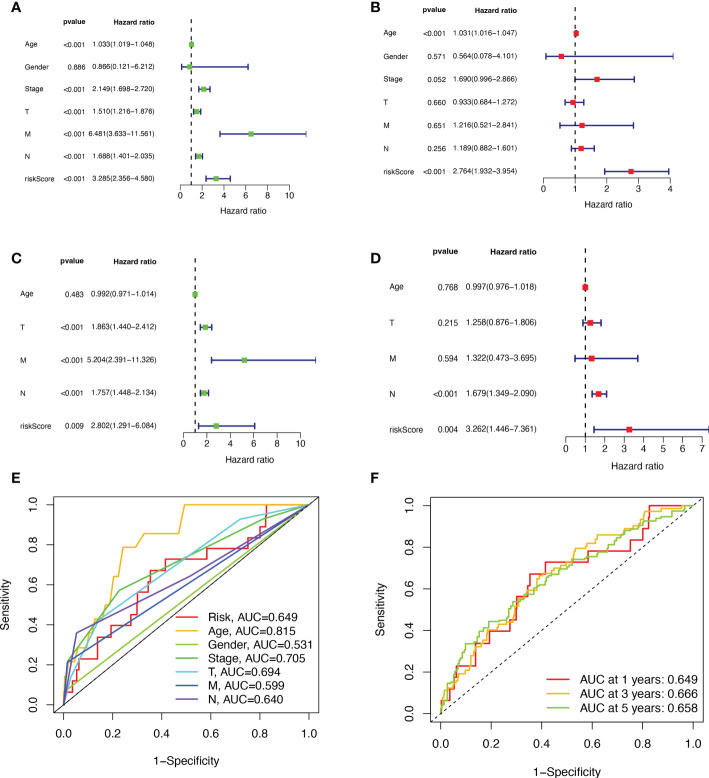
Validation of BMRGs prognosis and association with clinical parameters. **(A, B)** Univariate **(A)** and multivariate **(B)** Cox regression analyses of risk scores and clinical parameters in the training set (TCGA cohort). **(C, D)** Univariate **(C)** and multivariate **(D)** independent Cox regression analyses of risk scores and clinical parameters in the validation set (GEO cohort). **(E)** ROC curves and AUC for risk scores and clinicopathological characteristics. **(F)** ROC curves and AUC for 1-year, 3-year, and 5-year survival rates.

First, we preliminarily extracted 11 BMRGs that were highly correlated with BC prognosis using univariate Cox regression analysis. **Figure 1C** is a forest plot showing the difference in expression (*p* value) and hazard ratio (HR) of 11 BMRGs between cancer and normal tissues. We next penalized the 11 BMRGs by LASSO regression analysis (**Table 1**), whose curve is shown in **Figure 1D**. At last, we set up the final prognostic model by multivariate Cox regression analysis. The 7 best BMRGs (FBLN5, ITGB2, LAMC3, MMP1, EVA1B, SDC1, UNC5A) were included in the model analysis (**Table 2**) and their expression differences (*p* value) and hazard ratio (HR) are presented in **Figure 1E**. We calculated the risk score of each patient based on the regression coefficient and expression level of each gene, and divided the patients into high-risk and low-risk groups using the median risk score as the cut-off value. The survival curves in **Figures 2A, B** show that the difference in overall survival (OS) between the two groups was significant in the training and validation sets. The survival rate of the high-risk group in BC patients was significantly lower than that of the low-risk group, which initially confirms the predictive ability of our constructed model in terms of the prognosis of BC patients. **Figures 2C, D** demonstrated the distribution of risk score rankings of BC patients determined based on the seven BMRGs prognostic markers. The scatter plots of the training and validation sets in **Figures 2E, F** showed that the mortality rate of BC patients was positively correlated with the risk score. Survival time was negatively correlated with risk score. We plotted the heat map of **Figures 2G, H** to compare the expression levels of genes between high-risk and low-risk groups for the training and validation sets, from which it is known that in two different sets, the expression tendency of genes SDC1, ITGB2, MMP1, LAMC3 and UNC5A were basically the same, while the expression tendency of EVA1B and FBLN5 were different, as EVA1B was highly expressed in the low-risk group and lowly expressed in the high-risk group for the training set but it was highly expressed in both high-risk and low-risk groups for the validation set. And meanwhile, FBLN5 was almost lowly expressed in two groups for the training set and almost highly expressed in two groups for the validation set. Considering that the actual clinical conditions of patients are often complex, the prognosis is also influenced by many factors. We performed univariate and multivariate Cox regression analyses on the two datasets based on risk scores and clinical parameters. We drew forest plots (**Figures 3A-D**), respectively, to further confirm whether prognostic features could be used as independent prognostic indicators for BC patients. The univariate Cox regression analysis on the basis of the TCGA dataset showed that all factors, including age, tumor stage, T, N, and M stages, and model, except gender, could be used as independent prognostic indicators for BC patients (**Figure 3A**, *p*<0.001). And multivariate COX regression analysis showed that age and model were independent prognostic factors for BC patients (**Figure 3B**, *p*<0.001). Univariate COX regression based on the GEO dataset showed that T, M, and N stages could be independent prognostic indicators for BC patients (**Figure 3C**, *p*<0.001). Multivariate COX showed that N stage and model could be used as independent prognostic factors for BC patients (**Figure 3D**, *p*<0.005). Finally, we judged the reliability of the prognostic model by plotting the ROC curve and calculating the area under the curve (AUC). The AUC value of the risk score was 0.649, which had better predictive performance than the gender, M stage and N stage (**Figure 3E**). Our AUC values for predicting 1-year, 3-year, and 5-year survival were 0.649, 0.666 and 0.658, respectively (**Figure 3F**), which indicated that the prognostic model could effectively predict patient prognosis.

**Table 1:** The 11 basement membrane-associated genes obtained by LASSO regression analysis and their regression coefficients.

id	coef
CTSA	0.00779549049557869
FBLN5	-0.0162876081849546
FREM1	-0.160643851315573
ITGB2	-0.0107094447384872
LAMC3	0.204370545417446
MMP1	0.00324834178810158
EVA1B	-0.012127407265769
ITGAX	-0.0524544206014349
PAPLN	-0.0186576461581964
SDC1	0.00176256881594049
UNC5A	0.0409586439458294

LASSO, least absolute shrinkage and selection operator.

**Table 2:** The 7 basement membrane-associated genes obtained by multivariate Cox regression analysis and their regression coefficients.

id	coef
FBLN5	-0.029541632710079
ITGB2	-0.0235801263095758
LAMC3	0.249435180710723
MMP1	0.00387076833795619
EVA1B	-0.0146037458342459
SDC1	0.00213593388985154
UNC5A	0.0472256273299147

### Analysis of nomograms and biological pathways

**Figure 4:**
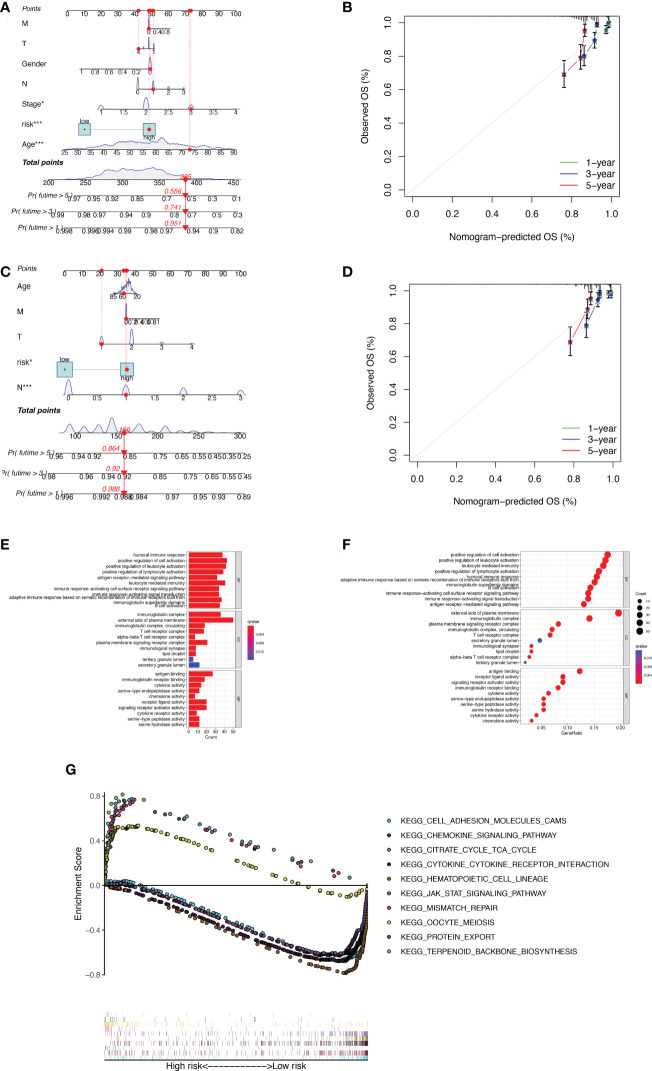
Construction of column line graphs and enrichment analysis of BMRGs. **(A–D)** Combined risk score and clinical parameters to predict the column line graphs of 1, 3, and 5-year OS of BC patients in the training set **(A)** and validation set **(C)**; calibration curves of 1, 3, and 5-year OS in the training set **(B)** and validation set **(D)**. **(E–F)** GO functional enrichment analysis. **(G)** GSEA multi-pathway enrichment analysis.

The nomogram was used to quantitatively predict the 1-, 3-, and 5-year OS of BC patients, which fully integrated risk scores and clinicopathological characteristics. **Figures 4A, B** exhibited the results about training set, we randomly selected one BC patient for scoring (**Figure 4A**). The calibration curves showed good agreement between the predictions of the column line graphs for 1-, 3-, and 5-year OS and the actual observed values (**Figure 4B**). The same results appear in the validation set (**Figures 4C, D**). To explore the biological process of BMRGs, we performed The Gene Ontology (GO) enrichment analysis and GSEA analysis. GO analysis showed that BMRGs were significantly associated with leukocyte mediated immunity, humoral immune response, and B cell activation (**Figures 4E, F**). GSEA enrichment analysis in **Figure 4G** showed that terpenoid backbone biosynthesis, citrate cycle TCA cycle, protein export, and mismatch repair were mainly enriched in the high-risk group. Hematopoietic cell lineage was more enriched in the low-risk group. Meanwhile, signaling pathways were more active in the low-risk group, such as JAK-STAT signaling pathway and CHEMOKINE signaling pathway.

### Tumor mutational burden

**Figure 5:**
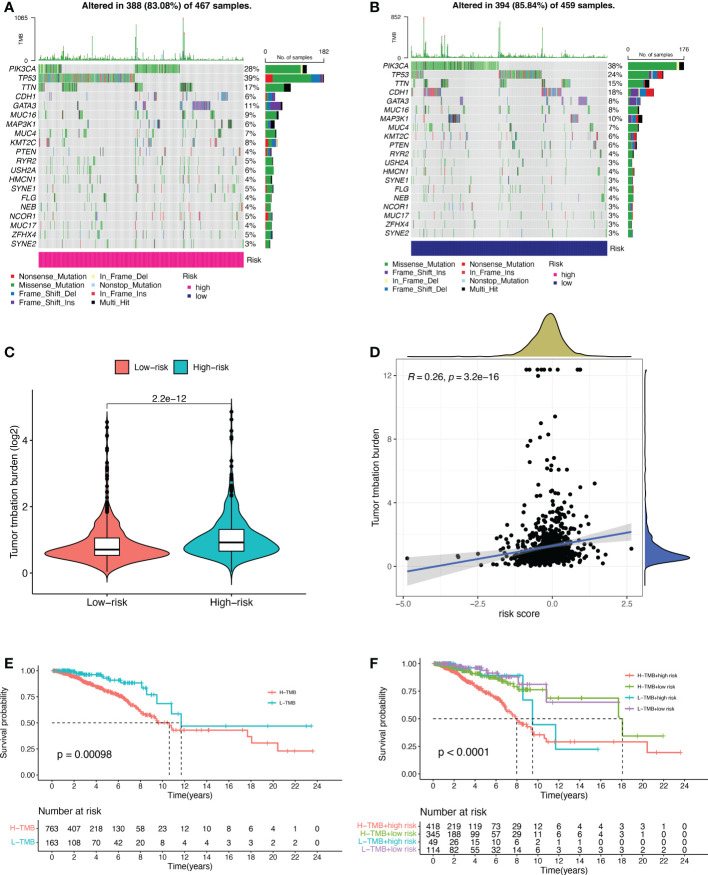
Tumor mutational burden (TMB) for prognostic features. **(A, B)** Waterfall plots of TMB in BC patients in the high-risk group **(A)** and low-risk group **(B)**. **(C)** Comparison of TMB between the high-risk and low-risk groups. **(D)** Correlation curves between TMB and risk scores. **(E)** Stratified survival curves of TMB in BC patients. **(F)** Survival curves for BC patients in terms of L-TMB + low risk, L-TMB + high risk, H-TMB + low risk, and H-TMB + high risk. H, high; L, low.

Based on the data from the TCGA dataset, we obtained theb mutations in BC patients using the R package “maftools”, which included a total of 926 BC samples. The mutation rate in the high-risk group was 83.08% (**Figure 5A**), and the mutation rate in the low-risk group was 85.84% (**Figure 5B**). From the waterfall plot, the mutated genes in the high-risk and low-risk groups were mainly PIK3CA, TP53, TTN, CDH1, GATA3, MUC16, MAP3K1, MUC4, KMT2C, and PTEN. These genes had different mutation rates in the low- and high-risk groups. The mutation rates of PIK3CA, CDH1, MAP3K1 and PTEN in the low-risk group were higher than those in the high-risk group. TMB was significantly higher in the high-risk group than in the low-risk group (**Figure 5C**, *p* = 2.2e-12). The correlation curve from **Figure 5D** showed a significant positive correlation between TMB and risk score (R = 0.26, *p* = 3.2e-16). The TMB survival curve in **Figure 5E** illustrates that patients with low TMB have a better prognosis (*p* = 0.00098). Among all groups in **Figure 5F**, BC patients with low risk + low TMB had the best prognosis when OS was less than 10 years.

### Tumor immune signature analysis

**Figure 6:**
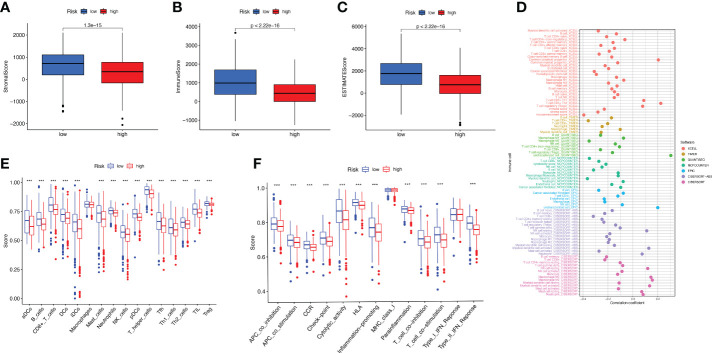
Study of BC immune status. **(A–C)** Box plots of StromalScore **(A)**, ImmuneScore **(B)**, and ESTIMATEScore **(C)** for high- and low-risk groups of BC patients. **(D)** Multiple algorithms to analyze immune infiltrating cells in BC. **(E, F)** Immune infiltrating cells **(E)** and immune-related function **(F)** scores for high- and low-risk groups.

As can be clearly seen from the boxplots of **Figures 6A-C** for tumor microenvironment scores, the StromalScore (*p* = 1.3e-15), ImmuneScore (*p*< 2.22e-16) and ESTIMATEScore (*p*< 2.22e-16) were significantly lower in the high-risk group than in the low-risk group. We tested the proportion of immune infiltration using a combination of algorithms. According to **Figure 6D**, the negative correlation coefficients were much larger than the positive correlation ones, suggesting that immunosuppressed patients tend to have higher classification indices. Common lymphoid progenitor, T cell CD4+ Th1, T cell CD4+ Th2 and T cell regulatory (Tregs) in XCELL, uncharacterized cell in QUANTISEQ, uncharacterized cell in EPIC, NK cell resting, Myeloid dendritic cell activated and Neutrophil in CIBERSORT-ABS, NK cell resting, Macrophage M0, Macrophage M2, Myeloid dendritic cell activated, Mast cell resting and Neutrophil in CIBERSORT were negatively correlated with the classifier index, while the other cells calculated using different algorithms cells were positively correlated with the classifier index. We determined the immunosuppression status and survival disadvantage of high-risk patients in our previous analysis. In addition, we performed single sample gene set enrichment analysis (ssGSEA) enrichment scores and on this basis further evaluated the relationship between risk scores and different immune cell subpopulations and functions. For the results, we found that ssGSEA scores of immune-related cells were significantly higher in almost all patients in the low-risk group (**Figure 6E**). Similarly in **Figure 6F**, APC co inhibition, APC co-stimulation, CCR, check point, cytolytic activity, HLA, inflammation promoting, parainflammation, T cell co inhibition, T cell co-stimulation, and Type Il IFN Response were all higher than in the high-risk group. The elevations of these items reflect the higher immune activity in the low-risk group.

### Consensus clustering of BMRGs identified two BC patient clusters

**Figure 7:**
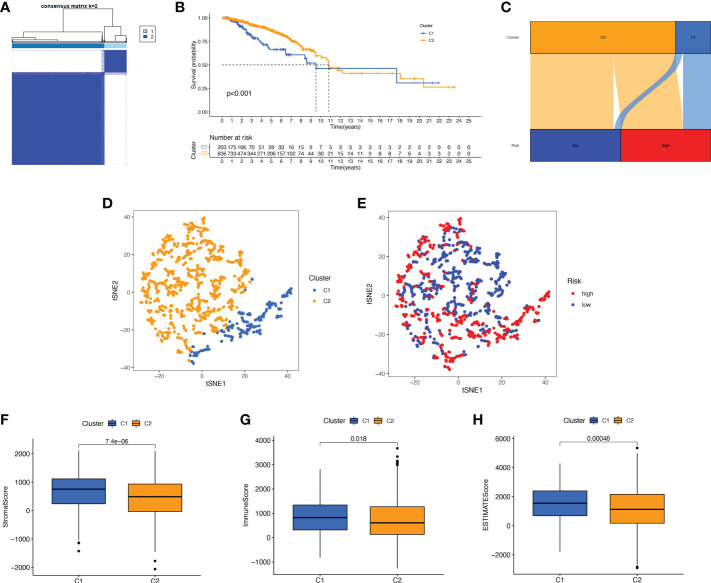
Survival, tSNE, and tumor microenvironment analysis for different clusters. **(A)** The best consensus matrix at k = 2. **(B)** Survival curves of BC patients in the two clusters. **(C)** Sankey diagram showing the distribution of risk scores between different clusters. **(D)** tSNE analysis of the two different clusters. **(E)** tSNE analysis of the high- and low-risk groups. **(F–H)** Box plots of StromalScore **(F)**, ImmuneScore **(G)**, and ESTIMATEScore **(H)** in different clusters. tSNE, t-Distributed Stochastic Neighbor Embedding.

**Figure 8:**
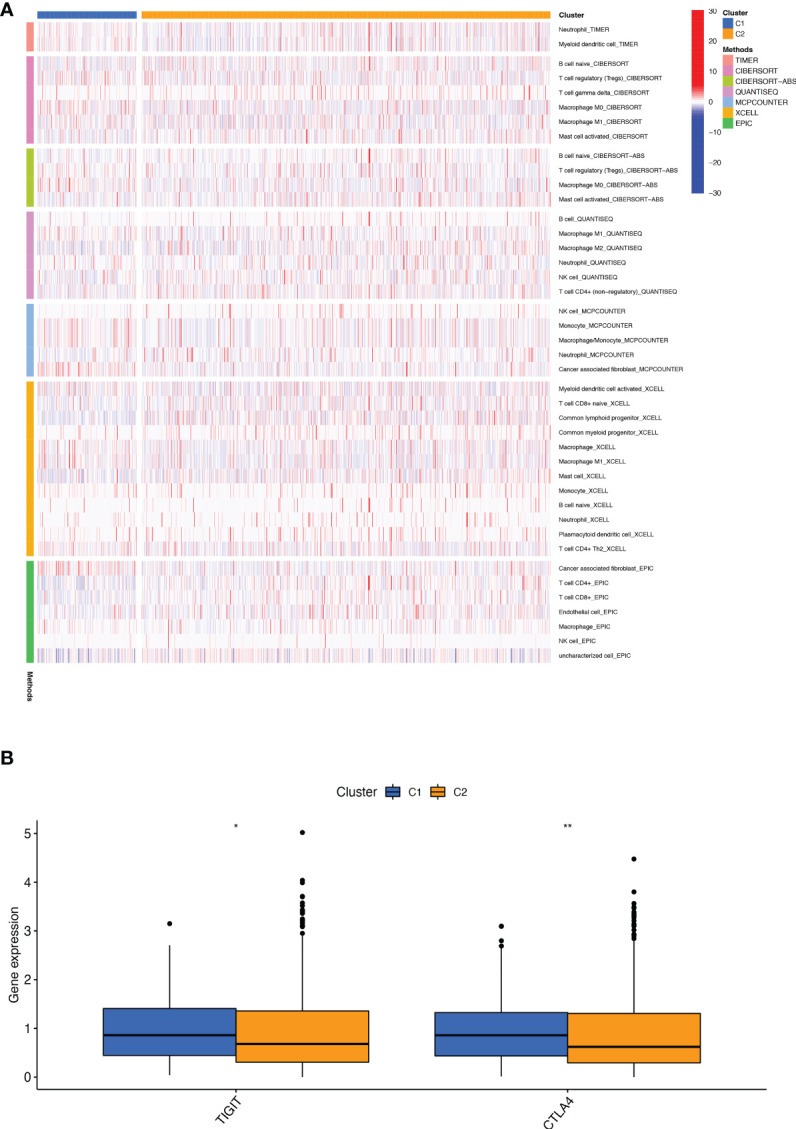
Infiltration and expression of immune cells, immune checkpoints in different clusters. **(A)** Analysis of infiltrating cells in two different clusters by using multiple algorithms. **(B)** Differential expression analysis of 2 immune checkpoint genes in different clusters. *p < 0.05; **p < 0.01.

We performed a consistent clustering analysis of the expression levels of seven BMRGs so as to investigate the relationship between these genes and breast cancer development. By shifting the clustering values from k = 2 to 9 respectively, we found that k = 2 had the strongest intra-cluster relationship. All breast cancer samples could be divided into 2 clusters on the basis of 7 genes (**Figure 7A**). The OS of patients in both clusters was measured by KM survival analysis and was calculated to be significantly different (**Figure 7B**, *p*<0.001). As seen in **Figure 7C**, the high-risk patients in cluster 1 accounted for the vast majority, and the number of low-risk patients in cluster 2 was predominant. **Figures 7D, E** show by t-SNE analysis that there are different dimensions between both different clusters and high and low-risk groups. Because of the tumor microenvironment’s important role in assessing prognosis, we also analyzed it. As can be seen, there was a significant difference between cluster 1 and cluster 2 (*p* = 7.4e-0.6) in StromalScore (**Figure 7F**). The same results occur in ImmuneScore (**Figure 7G**, *p* = 0.018) and in ESTIMATEScore (**Figure 7H**, *p* = 0.00046). Cluster 2 had significantly lower StromalScore, ImmuneScore and ESTIMATEScore than cluster 1. The immune response heat map was used to visualize the immune cell infiltration between the two clusters, indicating that cluster 2 had the most immune cell infiltration (**Figure 8A**). Given the importance of immunotherapy in tumor treatment, we went on to investigate the differences in immune checkpoint molecule expression between the different clusters. According to **Figure 8B**, the expression of 2 immune checkpoint molecules was significantly different between clusters. Both TIGIT and CLTLA4 had significantly higher expression in cluster 1 than those in cluster 2.

**Figure 9:**
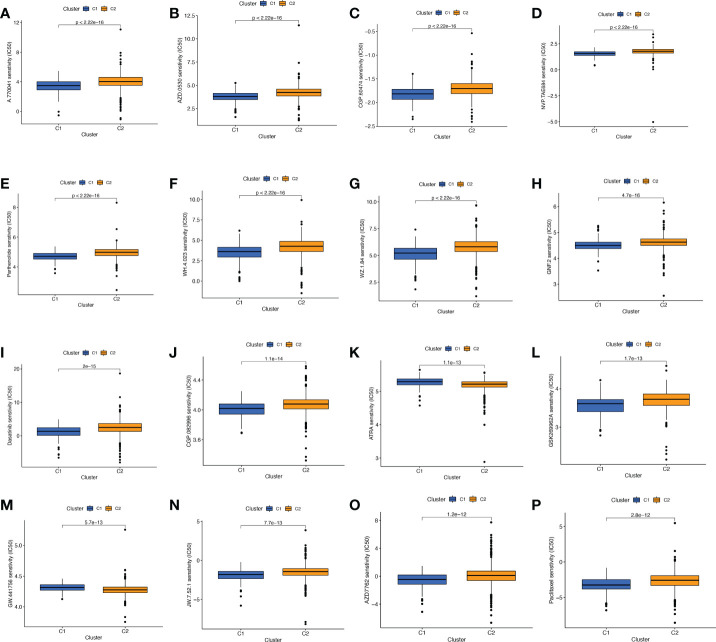
Comparison of potential therapeutic drug sensitivity between two different clusters. **(A)** A.770041. **(B)** AZD.0530. **(C)** CGP.60474. **(D)** NVP.TAE684. **(E)** Parthenolide. **(F)** WH.4.023. **(G)** WZ.1.84. **(H)** GNF.2. **(I)** Dasatinib. **(J)** CGP.082996. **(K)** ATRA. **(L)** GSK269962A. **(M)** GW.441756. **(N)** JW.7.52.1. **(O)** AZD. **(P)** Paclitaxel. The top 16 with the lowest p value were showed.

Screening of sensitive drugs is the foundation for the clinical treatment of tumors. Drug sensitivity was assessed using IC50, and **Figures 9A-P** showed the 16 drugs with the most significant differences in sensitivity. We can see that BC patients in Cluster 1 were sensitive to A.770041 (Lck inhibitor), AZD.0530 (Saracatinib), CGP.60474 (CDK inhibitor), NVP.TAE684 (ALK inhibitor), Parthenolide, WH.4. 023 (Src inhibitor), WZ.1.84, GNF.2 (Bcr-Abl inhibitor), Dasatinib, CGP.082996 (CDK4 inhibitor), GSK269962A (ROCK inhibitor), JW.7.52.1 (mTOR inhibitor), AZD7762 (Chk1 inhibitor), and Paclitaxel. The patients in cluster 2 were more sensitive to ATRA (All-Trans Retinoic Acid) and GW.441756 (TrkA inhibitor). We can clearly conclude that BC patients in cluster 1 have more sensitive clinical agents to develop than those in cluster 2.

### Clinical drug sensitivity analysis and immunotherapy efficacy evaluation of prognostic characteristics of BMRGs

**Figure 10:**
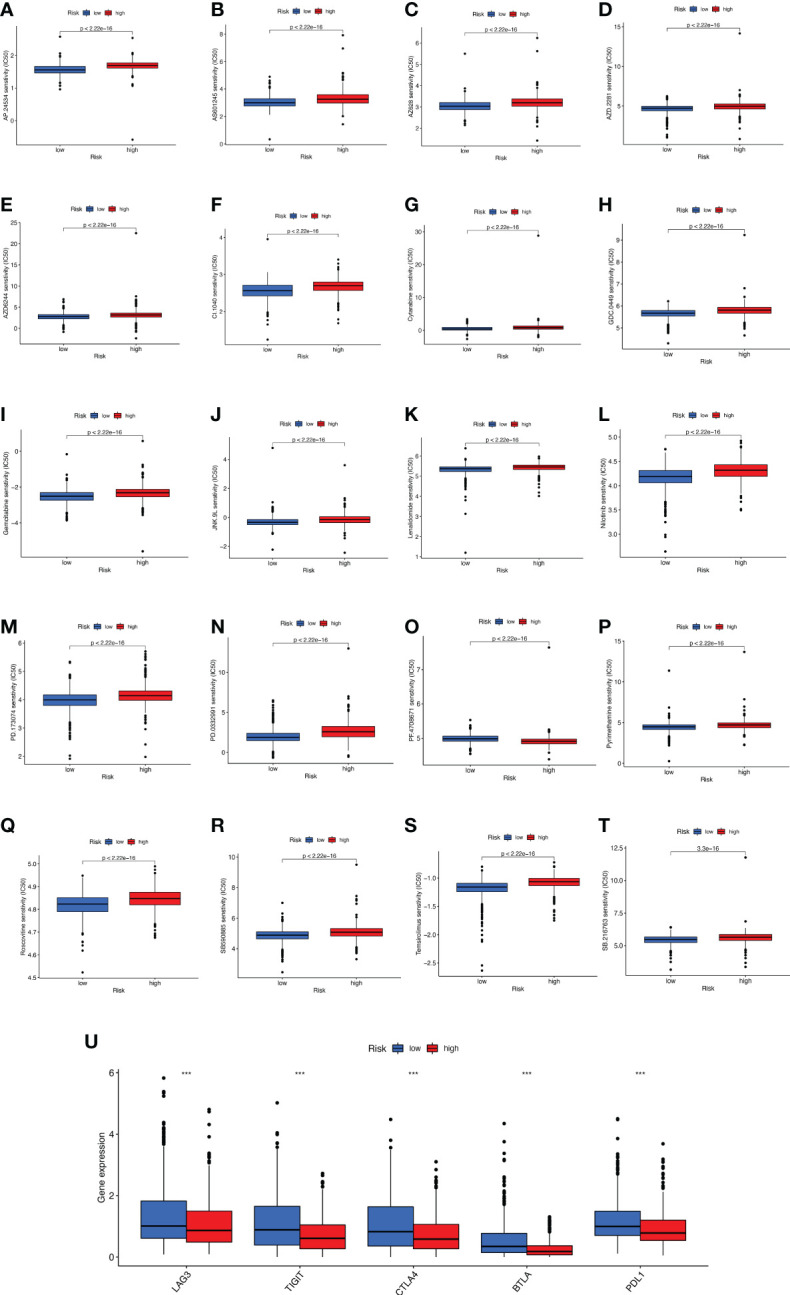
Analyses of potential drug sensitivity and immune checkpoint gene expression between high- and low-risk groups. **(A–T)** The boxplots for drug sensitivity analysis of **(A)** AP.24534. **(B)** AS601245. **(C)** AZ628. **(D)** AZD.2281. **(E)** AZD6244. **(F)** CI.1040. **(G)** Cytarabine. **(H)** GDC.0449. **(I)** Gemcitabine. **(J)** JNK.9L. **(K)** Lenalidomide. **(L)** Nilitinib. **(M)** PD.173074. **(N)** PD.0332991. **(O)** PF.4708671. **(P)** Pyrimethamine. **(Q)** Roscovitine. **(R)** SB590885. **(S)** Temsirolimus. **(T)** SB.216763. **(U)** Immune checkpoint genes differential expression analysis. ***p < 0.001.

The promising research related to drug treatment of BC is a hot topic of current research and has received widespread attention. We analyzed the relationship between risk scores and drug resistance by calculating the IC50 of drugs against BC. The 20 most significantly different drugs are listed in **Figure 10**. We noted that PF.4708671 (S6 Kinase inhibitor) had a higher IC50 in high-risk patients, while all the rest of the other drugs had a higher IC50 in low-risk patients (**Figures 10A-T**). Considering the broad clinical application of ICI, we further investigated the expression of genes at immune checkpoints between the 5 high and low-risk groups. **Figure 10U** shows that the gene expression levels of immune checkpoints were lower in all high-risk groups than in the low-risk group, and the results were significant (*p*<0.001).

## Discussion

BC is one of the most common malignancies in the world and the leading cause of most cancer-related deaths (25). Despite significant advances in early diagnosis and treatment strategies in recent decades, the prognosis of BC remains poor. BM is an extracellular matrix that adheres to the underside of epithelial cells. Its core structural components, type IV collagen, laminin, nidogens, and heparan sulfate proteoglycans, are essential in directing cell polarity, differentiation, migration, and survival (3). It has been shown that disruption of BM structure and loss of components are associated with tumor invasion and metastasis and are considered as potential indicators of cancer progression with good predictive power in terms of diagnosis and prognosis. This study is the first to elaborate a BC model of Basement membrane related genes (BMRGs) in terms of survival prognosis, gene enrichment analysis, molecular typing, tumor immune microenvironment, and drug sensitivity analysis. In this study, we constructed a reliable prognostic signature to provide a new direction for further study of individualized treatment strategies and prognosis prediction for BC patients.

This study screened the 7 best BMRGs (FBLN5, ITGB2, LAMC3, MMP1, EVA1B, SDC1, UNC5A) to construct a predictive model. Previous studies have shown that basement membrane-associated genomes are associated with other BC-related pathophysiology. ITGB2-AS1 has a facilitative effect on BC cell migration and invasion through the upregulation of ITGB2 (26). SDC1 is a heparan sulfate proteoglycan (27). SDC1 overexpression in BC was found to promote cancer cell growth and proliferation and was associated with the methylation status of the SDC1 promoter (28). SDC1 may also promote BC cell migration across the blood-brain barrier BBB by regulating the cytokines of the blood-brain barrier (BBB) (29). Y-box binding protein-1 (YB-1) increases BC cells’ invasive and metastatic ability through the upregulation of MMP1 (29). UNC5A was reported to be lowly expressed in BC and is associated with a poor prognosis of BC (30). Silencing of UNC5A during BC progression may be related to mutation and DNA methylation (31). In addition, in BC cells and tumor tissues, FBLN5 expression downregulates the activation of the NF-κB signaling pathway to promote BC cell proliferation and migration (32). Studies have shown that EVA1B expression is upregulated in colon cancer and glioma (33, 34). However, few EVA1B-related studies have been found in BC. LAMC3 gene encodes the laminin γ-3 chain (35). Low expression of LAMC3 may be associated with poor prognosis and malignant progression in OC of ovarian cancer (36). Although the mechanisms of EVA1B and LAMC3 are currently little studied in BC, their role in other tumors can be identified, suggesting that more relevant studies should be done in BC. Taken together, these BMRGs in associated with BC development and progression. Therefore, a prognostic model constructed based on the seven BMRGs mentioned above seems reliable.

We assessed the prognostic value of BMRGs by training and validating survival curves, risk score plots, survival status plots, and heat maps. The scatter plots of the training and validation sets showed that the survival status of BC patients was related to the risk score, and the mortality of patients increased with the risk score. Combining the seven prognostic BMRGs, we plotted heat maps to compare the expression levels of genes in the training and validation sets in the high-risk and low-risk groups, and it could be seen that the expression trends of LAMC3, MMP1, SDC1, UNC5A and ITGB2 were consistent. In contrast, the expression trends of EVA1B and FBLN5 were inconsistent. We speculated that there might be two reasons for the different gene expression trends. The training and validation sets were from two databases (TCGA and GEO). On the one hand, the sample sizes screened by the two databases are different, and the different sample sizes might impact the percentage of their gene expressions. On the other hand, the two databases were from diverse populations. The TCGA data were from Europe and the US, while the GEO data were from Taiwan. The number, ethnicity, tissues taken, and age group might influence the percentage of gene expression. Our findings have clinical and pathological implications. Univariate and multivariate analyses showed that risk score could be an independent prognostic factor for BC patients (p<0.001). The AUC values of 0.649 for prognostic features and 0.649, 0.666, and 0.658 for 1-year, 3-year, and 5-year predicted survival, respectively, showed not only better predictive performance compared with other clinicopathological features (sex, stage, T-stage, N-stage, and M-stage), but also reflected the reliability and accuracy of prognostic features.

Through GO enrichment analysis, we noted that leukocyte-mediated immunity, humoral immune response, and B cell activation play critical roles in the biological pathways associated with BMRGs. Through GSEA enrichment analysis, we found that high-risk patients have abundant biosynthetic and metabolic pathways, such as terpenoid backbone biosynthesis, citrate cycle, and protein export. Undoubtedly, tumor development is dependent on high levels of biosynthesis and metabolism. In contrast, the hematopoietic cell lineage pathway is more active in low-risk patients. The establishment of hematopoietic and immune capacity inhibits the pro-carcinogenic effect. erk1/1-mediated CD44 expression increases stromal production and migration, leading to stromal expansion (37). cd44 and its new ligand, serum glycine, a hematopoietic cell lineage-specific proteoglycan, may be jointly involved in lymphocyte adhesion and activation (38). It can be speculated that BC in the low-risk group is more likely to suppress tumor progression by establishing immunity, especially the mediation of leukocyte, B cell and the involvement of hematopoietic cell lineage pathways, which deserve further exploration as it could provide new strategies for immunotherapy studies.

TMB indicates the number of mutations per megabase (Mut/Mb) in DNA sequenced in cancers (39). Studies have shown that TMB is considered a reliable predictive biomarker for the efficacy of cancer patients after taking ICI therapy (40–42). In the TCGA cohort, we found a significant correlation between TMB and risk score. High-risk patients had a higher TMB and poorer OS. Low-risk patients had a lower TMB and better OS. The highest mutation rate of the TP53 gene in high-risk patients is consistent with previous reports that mutations in the TP53 gene were frequently detected in high-risk BC patients (43, 44). TP53 gene is an important oncogene, and its conversion is associated with moderate to high risk, poor outcome, and poor prognosis in BC (45, 46). And the highest mutation rate of the PIK3CA gene was found in low-risk patients.

Immunotherapy has emerged as the most promising approach for the treatment of cancer, and the effectiveness of immunotherapy is closely related to immune infiltration (47). We performed ssGSEA enrichment scoring in the TCGA and GEO cohorts and found that the infiltration of immune cells was significantly higher in patients in the low-risk group than in the high-risk group. It was shown that the interaction of BC cells with BM affects tumor progression by influencing immune cells in the tumor microenvironment (48). In addition, immune functions such as APC co-stimulation, T cell co-stimulation, and Type Il IFN Response activity were significantly higher in the low-risk group than in the high-risk group. APC antigen presentation activates T cells, which secrete type II interferon (IFN) - γ. Studies have shown that T cells play an essential role in anti-tumor *in vivo* (49). In summary, our risk model based on BMRGs is closely related to the tumor immune microenvironment.

Next, we divided BC patients into 2 clusters to explore the relationship between tumor subtypes. Cluster 2 had a high proportion of low-risk patients with the highest immune scores and the most immune cell infiltration, indicating that cluster 2 was the most immunologically active. The study showed that the immune cells with the highest degree of infiltration in cluster 2 were CD8+ T cells. According to St Paul M et al., CD8+ T cells produce IFN-γ to exert their cytotoxic effects to kill cancer cells (50). It can be speculated that BC patients in cluster 2 may have better clinical immunotherapy outcomes. Furthermore, we found that the immune checkpoint molecules TIGIT and CLTLA4 were highly expressed in cluster 1 and that high-risk patients accounted for many in cluster 1. Recently, significant progress has been made in immunotherapy targeting immune checkpoint molecules (51). The above results suggest that BC patients in the high-risk group may benefit from immunotherapy related to the two immune checkpoint molecules.

Investigating the relationship between high and low risk and the sensitivity of molecularly targeted drugs and chemotherapeutic agents is essential. We found that BC patients in the high-risk group were most sensitive to all-trans retinoic acid (ATRA) and TrkA inhibitor. one of the principles of the antitumor activity of ATRA is that it is a promising agent for the prevention/treatment of BC by reducing the number of mitochondria leading to an inadequate respiratory/energy balance in BC cells (52). The combination of ATRA with protein kinase Cα/β1 inhibitors has been shown to inhibit breast tumor progression in hormone-non-dependent BC models. However, the antitumor effects in solid tumors still need further investigation (53). *In vitro* experiments have shown that TrkA overexpression enhances BC cell growth and invasion by activating Erk1/2 and PI3K-AKT-mediated signaling pathways (54). Functional experiments have demonstrated that TrkA inhibitor reduces cell viability by decreasing phosphorylated TrkA and downstream AKT, providing therapy for HER2-positive BC (55). For those drugs that have not yet been applied in the clinic, our study provides some theoretical basis for their development. The above results suggest that, on the one hand, risk assessment of tumors may be a potential predictor to guide targeted therapies and immunotherapy in BC patients; on the other hand, BM may be of high research value in the development of BC at intermediate and late stages.

Oncology therapy is an essential area of interest. Through IC50 screening analysis of potential drugs, we found that high-risk patients may be sensitive to PF.4708671, the first reported selective inhibitor of S6K1 (56). Choi HN showed that the highly specific inhibitor PF-4708671 enhances glucose deprivation-induced apoptosis by downregulating anti-apoptotic proteins in BC cells (57). PF-4708671 inhibits S6K1, the most downstream kinase in the mTOR pathway, to inhibit BC cell migration in a triple-negative BC metastasis model and thus may provide an effective adjuvant treatment against BC metastasis (58). However, patients at high risk of BC are not susceptible to AP.24534 (Ponatinib), AS601245 (JNK inhibitor), AZ628 (Raf inhibitor), AZD.2281 (Olaparib), AZD6244 (Selumetinib), CI.1040 (Mek inhibitor), Cytarabine, GDC.0449 (Vismodegib), Gemcitabine, JNK.9L, Lenalidomide, Nilotinib, PD.173074 (FGFR inhibitor), PD.0332991 (Palbociclib), PF.4708671 (S6 Kinase inhibitor), Pyrimethamine, Roscovitine (CDK inhibitor), SB590885 (B-Raf inhibitor) and Temsirolimus may have resistance. Studies have shown that the combination of multiple drugs with different molecular mechanisms can significantly improve the resistance problems caused by single mechanism alterations, thus ensuring the efficacy of the drugs (59–61). In addition, ICIs are one of the successful immunotherapy strategies for triple-negative breast cancer (TNBC) (62, 63). A growing body of data suggests that monoclonal antibodies against PD-1/PD-L1, a type of ICI, can induce durable clinical responses in some patients with metastatic TNBC and may also have meaningful clinical activity in patients with rare ER+ HER-2- BC (64). On the one hand, we believe that the prognostic characteristics of BMRGs can be used as a new indicator to assess ICI. On the other hand, we identified a series of potential, worthwhile drugs for BC patients. At the same time, the actual effects of these drugs need to be further demonstrated in more clinical trials.

Beyond all doubt, our study has the following limitations. First, although we have validated our prognostic risk scores in the TCGA and GEO datasets, validation of differentially trending expressed genes in an expanded sample size may further increase the confidence of the risk scores. Second, we confirmed that the prognostic features of BMRGs have good predictive value, but *in vivo* and *in vitro* experiments are still needed to reveal the role of BMRGs in the onset and progression of BC. Third, we still need more clinical data and prospective studies to validate the clinical value of the BMRGs prognostic signature.

## Conclusion

We constructed and validated a model for the prognostic characteristics of BMRGs, which includes FBLN5, ITGB2, LAMC3, MMP1, EVA1B, SDC1, UNC5A. The signature can be used as an independent prognostic factor for BC and can provide a new direction for individualized treatment of BC patients.

## Data availability statement

The original contributions presented in the study are included in the article/**Supplementary Material**. Further inquiries can be directed to the corresponding author.

## Author contributions

JC, XZ, and AL conceived and designed the study. WX and ZL involved themself in data collection, collation, and statistical analysis. WL ran the R language and drew and analyzed the graphs. XZ, WX, and AL participated in the literature search and revised the manuscript for important content. JC, ZL and AL analyzed, interpreted the results, and edited a draft version of the manuscript. All authors contributed to the article and approved the submitted version.

## Funding

This work was supported by the Special Fund Project of Guangdong Science and Technology (210728156901524), Shantou Medical Science and Technology Planning Project (grant number 200622115260639,2021-68-44,2022-88-27), Administration of Traditional Chinese Medicine of Guangdong Province project (202205092315428030), the Scientific Research Fund of Hunan Provincial Education Department (No. 20A213 and No. 19B236) and Natural Science Foundation of Hunan Province (No. 2020JJ5221).

## Acknowledgments

This is a short text to acknowledge the contributions of specific colleagues, institutions, or agencies that aided the efforts of the authors.

## Conflict of interest

The authors declare that the research was conducted in the absence of any commercial or financial relationships that could be construed as a potential conflict of interest.

## Publisher’s note

All claims expressed in this article are solely those of the authors and do not necessarily represent those of their affiliated organizations, or those of the publisher, the editors and the reviewers. Any product that may be evaluated in this article, or claim that may be made by its manufacturer, is not guaranteed or endorsed by the publisher.
